# Postnatal symptomatic Zika virus infections in children and adolescents: A systematic review

**DOI:** 10.1371/journal.pntd.0008612

**Published:** 2020-10-02

**Authors:** Anna Ramond, Ludmila Lobkowicz, Nuria Sanchez Clemente, Aisling Vaughan, Marília Dalva Turchi, Annelies Wilder-Smith, Elizabeth B. Brickley

**Affiliations:** 1 Department of Infectious Disease Epidemiology, London School of Hygiene & Tropical Medicine, London, United Kingdom; 2 Instituto de Patologia Tropical e Saúde Pública, Universidade Federal de Goiás, Goiânia, Brasil (Institute of Tropical Pathology and Public Health, Federal University of Goias, Goiânia, Brazil); 3 Department of Disease Control, London School of Hygiene & Tropical Medicine, London, United Kingdom; 4 Heidelberg Institute of Global Health, University of Heidelberg, Heidelberg, Germany; 5 Department of Epidemiology and Global Health, Umeå University, Umeå, Sweden; Aix-Marseille Universite, FRANCE

## Abstract

**Background:**

Recent Zika virus (ZIKV) outbreaks in the Pacific and the Americas have highlighted clinically significant congenital neurological abnormalities resulting from ZIKV infection in pregnancy. However, little is known about ZIKV infections in children and adolescents, a group that is potentially vulnerable to ZIKV neurovirulence.

**Methods:**

We conducted a systematic review on the clinical presentation and complications of children and adolescents aged 0 to 18 years with a robust diagnosis of ZIKV infection. We searched PubMed, Web of Science, LILACs, and EMBASE until 13 February 2020 and screened reference lists of eligible articles. We assessed the studies’ risk of bias using pre-specified criteria.

**Findings:**

Our review collated the evidence from 2543 pediatric ZIKV cases representing 17 countries and territories, identified in 1 cohort study, 9 case series and 22 case reports. The most commonly observed signs and symptoms of ZIKV infection in children and adolescents were mild and included fever, rash, conjunctivitis and arthralgia. The frequency of neurological complications was reported only in the largest case series (identified in 1.0% of cases) and in an additional 14 children identified from hospital-based surveillance studies and case reports. ZIKV-related mortality was primarily accompanied by co-morbidity and was reported in one case series (<0.5% of cases) and three case reports. One death was attributed to complications of Guillain-Barré Syndrome secondary to ZIKV infection.

**Conclusions and relevance:**

Based on the current evidence, the clinical presentation of ZIKV infection in children and adolescents appears to be primarily mild and similar to the presentation in adults, with rare instances of severe complications and/or mortality. However, reliable estimation of the risks of ZIKV complications in these age groups is limited by the scarcity and quality of published data. Additional prospective studies are needed to improve understanding of the relative frequency of the signs, symptoms, and complications associated with pediatric ZIKV infections and to investigate any potential effects of early life ZIKV exposure on neurodevelopment.

## Author summary

Although the number of Zika virus (ZIKV) cases has declined following the 2015–2016 outbreak in the Americas, ZIKV remains a public health concern due to the potentially severe consequences of *in utero* exposure to the virus. While there is an increasing understanding of the effects of prenatal congenital ZIKV infection, less is known regarding the potential consequences of postnatal non-congenital ZIKV infection in children and adolescents. As this age group may also be vulnerable to the adverse effects of ZIKV on the nervous system, a better understanding of ZIKV infection in this population is needed. This knowledge may help to inform the case definition for ZIKV disease in children and elucidate whether children and adolescents should be included in strategies and measures for prevention of ZIKV infection, which are currently aimed primarily at pregnant women. After a review of the existing literature, the authors found that ZIKV infection in children and adolescents appears to be similar to adults and primarily mild, with little evidence of severe neurological consequences. Further research using prospective methods and neurodevelopmental assessments is warranted.

## Introduction

Following the introduction of the arthropod-borne flavivirus Zika (ZIKV) into Brazil in 2013 [1], ZIKV spread rapidly across the Americas, facilitated by global air travel [2], and autochthonous transmission of ZIKV has now been reported in 84 countries and territories worldwide [3]. After the explosive 2015–2016 outbreak in the Americas, a rapid decline in cases was observed from 2017 onwards [4]; however smaller clusters, often uncovered by travelers [5–8], continue to be detected beyond the Americas, in Asia [9–13] and Africa [14, 15].

Valuable progress has been achieved in recent years in terms of understanding the transmission and clinical presentation of ZIKV infections in adults and congenitally infected neonates. Although around 29–82% of ZIKV infections in the general population have been reported to be asymptomatic [16], the most frequently reported signs and symptoms include rash, fever, arthralgia, and conjunctivitis [3, 17]. Evidence has shown that ZIKV is also neurotropic, with approximately 5–10% of congenital ZIKV infections leading to neurologic anomalies in neonates [18–24]. A specific constellation of structural abnormalities and functional disabilities is now recognized as Congenital Zika Syndrome (ICD-10 P35.4). The definition includes five unique features (i.e., severe microcephaly, subcortical calcifications, macular scarring and pigmentary retinal mottling, congenital contractures, and early hypertonia), which are used to distinguish congenital ZIKV infection from other congenital infections [25, 26]. Another severe neurological complication which has been linked with ZIKV infection is the severe autoimmune polyneuropathy known as Guillain-Barré Syndrome (GBS) [27, 28].

While the presentation of ZIKV infection in adults is well-defined, there is limited information on the spectrum of clinical manifestations of ZIKV infections in children and adolescents (defined here as 0–18 years). This is an important gap to address as this age group comprises a substantial fraction of all ZIKV infections. Reports on the proportion of children and adolescents among ZIKV cases range from 10% in the U.S. (January—December 2016) [29] to 24% in Colombia (August 2015—April 2016) [30] and 31% in the state of Pernambuco in Brazil (November 2015—October 2016) [31]. Reports on the proportion of symptomatic ZIKV infections in children and adolescents range from 41% in Nicaragua [32] to 70% in French Polynesia [33]. Further, given the importance of brain development in childhood and adolescence, and the neurovirulence of ZIKV, children may be more susceptible to the adverse neurological consequences of ZIKV infection [34].

To our knowledge, no systematic review to date has examined the existing literature on the clinical presentation of ZIKV infections in children and adolescents. This study aims to give an overview of the spectrum of clinical manifestations of postnatal ZIKV infections in children and adolescents (0–18 years) and to highlight existing knowledge gaps for the scientific community to stimulate further research.

## Methods

### Search strategy and selection criteria

We conducted this systematic review following a pre-defined research protocol registered in the PROSPERO database (CRD42019119260), in accordance with PRISMA guidelines (S1 Table) [35]. To identify studies reporting on postnatal ZIKV infection in children, we performed a comprehensive literature search, with no date or language restrictions, using four databases (PubMed, Web of Science, LILACs, and EMBASE) from 1956 to 13 February 2020. A broad search strategy was used to identify studies reporting on postnatal ZIKV infection in children. The search strategy utilized Medical Subject Headings and keywords, including English, French, Spanish and Portuguese translations, relating to ZIKV and pediatric populations (S1 Methods). No date or language restrictions were applied.

### Study selection and data extraction

Eligible studies included cohort, cross-sectional, case series, and case report studies reporting on symptoms of postnatal ZIKV infection in children, aged 0 to 18 years of age, with a robust confirmation of ZIKV infection (i.e., laboratory confirmation by molecular (reverse transcription polymerase chain reaction [RT-PCR]) or serologic test (Immunoglobulin (Ig) M and G enzyme-linked immunosorbent assays [ELISAs], antibody hemagglutination assay, plaque reduction neutralization test [PRNT], or Council of State and Territorial Epidemiologists [CSTE] criteria [36]). Studies reporting on symptoms of congenital ZIKV infection or with evidence of a co-infection were excluded from the review. Three reviewers (AR, LL, and AV) screened identified studies for eligibility in duplicate; any discrepancies were resolved by a third reviewer (EBB and NSC). References of eligible studies were also screened to identify additional potentially relevant studies. Three reviewers (AR, LL, and AV) performed the data extraction independently and cross-verified the results for accuracy and consistency. Extracted data included information on: study author, study location, year, number of ZIKV cases, population source, ZIKV diagnostic testing, and differential diagnostic testing. Outcomes of interest extracted were the frequency of rash, fever, conjunctivitis (i.e., inclusive of other signs of conjunctival involvement, such as conjunctival hyperemia), arthralgia, myalgia, and headache and other signs and symptoms of ZIKV infection [3, 17]. For case reports, additional information on patient complications and co-morbidities were extracted where available. A world map indicating the location of studies was created using Tableau Desktop 2019.2.2.

### Quality assessment

Two reviewers independently assessed study quality. The cohort study was assessed using the Oxford Centre for Evidence-based Medicine (OCEBM) Levels of Evidence, March 2009, which range from level 1 (highest) to 5 (lowest level of evidence) [37]. The case series and case reports were evaluated using the study quality criteria proposed by Murad and colleagues (2018) [38]. Overall risk of study bias was based primarily on the assessment of participant exposure (i.e., ZIKV diagnosis) and outcomes (i.e., ZIKV signs, symptoms, and complications).

## Results

Our search returned a total of 9440 records, of which 2825 were duplicates and were removed (S1 Fig). After screening 6614 individual records, we removed 6394 by title or abstract. We assessed 220 full text articles for eligibility, of which 188 were not included due to: study design (reviews or commentary articles, n = 32; conference abstracts, n = 12), study outcome (no information on clinical signs, symptoms, or complications, n = 53), study sample (different target population, n = 56), and exposure ascertainment (lack of robust ZIKV confirmation, n = 6). We excluded an additional 30 studies, in which cases had congenital ZIKV infections (n = 23) or known co-existing infections with other pathogens (n = 7). We identified one additional full text article from the reference review of eligible articles. This resulted in 32 final full text articles included in the systematic review (i.e., 1 cohort, 9 case series and 22 case reports).

### Cohort and case series studies

**Fig 1:**
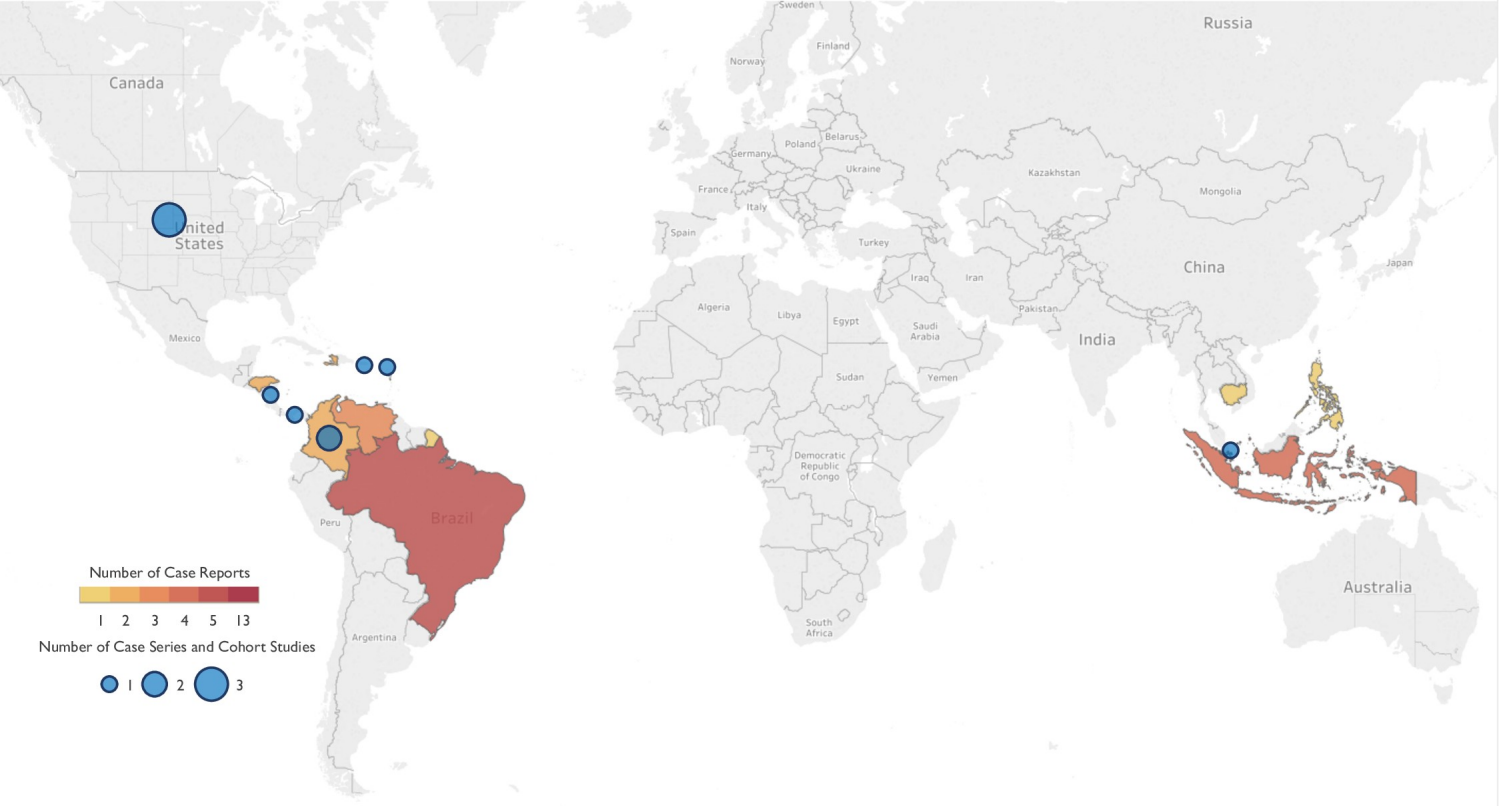
World map displaying the location of the 32 studies included in the systematic review. For studies reporting on travel-associated infections, the country of infection was reported as location of study. Dark red indicates the countries with the greatest representation of case reports. Size of blue circle indicates the number of cohort and case series studies within a given country.

Ten studies, including a total of 2503 cases and conducted across 7 countries and territories (Colombia, Guadeloupe, Panama, Puerto Rico, Singapore, Nicaragua and the United States of America, Fig 1), reported on the presence of ZIKV symptoms in a pediatric study population or sub-group (Table 1). Only one investigation characterized the clinical presentation of pediatric ZIKV cases in a prospective cohort study [39]. In eight studies, children and adolescent ZIKV cases were identified using ZIKV surveillance systems [40–47]. In five of these studies, data were obtained from national or county level surveillance systems [40, 41, 43, 45, 48], while passive surveillance and hospital-based surveillance systems were respectively used in the three remaining studies [44, 46, 47]. In the final case series, possible ZIKV cases were identified using physician reports [49]. According to the OCEBM Levels of Evidence, the one cohort study was graded Level 1b. Within the case series, the study quality varied, reflecting differences in population sampling and study design, with one case series rated good [47], five of the case series rated fair [41, 42, 44, 45, 49], and three studies rated poor [40, 43, 46] (S2 Table).

**Table 1:** Summary of cohort study and case series reporting on symptoms of postnatal ZIKV infection in children and adolescents, ordered by number of ZIKV cases.

Author (year)	Location	Study year	Case identification	Case definition for confirmatory testing	% Female	Median age in years (range)	ZIKV diagnostic test	N cases	Common signs and symptoms (%)						Additional signs, symptoms and complications (%)		
									Rash	Fever	Conjunctival involvement	Arthralgia	Myalgia	Headache	URT symptoms^a^	GI symptoms^b^	Neurological complications
Tolosa et al., (2017) [43]	Colombia	2015–2016	National surveillance system	National case definition^c^	72%	NR (1 month-18y)	RT-PCR	1207	NR	NR	NR	NR	NR	NR	NR	NR	1%
Burger-Calderon et al., (2020) [39]	Nicaragua	2016–2017	Prospective cohort study	Fever and/or rash, ± any other clinical finding	55%	8.7 (2–14)	RT-PCR or serological algorithm^d^	556	79%	60%	11%	25%	9%	37%	21%	2%	NR
Read et al., (2018) [44]	Puerto Rico	2012–2016	Hospital/ clinic-based surveillance system	Fever, or acute onset of rash, arthritis, arthralgia or conjunctivitis	46%	NR (0–18)	RT-PCR	351	80%	99%	58%	37%	37%	64%	38%	NR	NR
Goodman et al., (2016) [40]	United States of America	2016	National surveillance system	CSTE case definition^e^	56%	14 (1 month-17)	CSTE case definition^e^	158	82%	55%	29%	28%	NR	NR	NR	NR	0%
Lindsey et al., (2020) [45]	United States of America	2016–2017	National surveillance system	CSTE case definition^e^	64%	8.5 (0–17)	RT-PCR or IgM ± PRNT	140^f^	94%	74%	36%	48%	39%	50%	26%^g^	27%^h^	0%
Griffin et al., (2017) [41]	United States of America	2015–2017	County surveillance system	State testing criteria^i^	48%^j^	11 (1–17)^j^	RT-PCR or IgM and PRNT	33	94%	76%	27%	21%	6%	NR	21%	15%	0%
Cordel et al., (2017) [49]	Guadeloupe	2016	Clinic-based/medical records	Rash and WHO definition^k^	NR	7 (5)^l^	RT-PCR	29	100%	59%^m^	64%^m^	NR	NR	NR	NR	NR	NR
Ho et al., (2017) [42]	Singapore	2016	National surveillance system	Fever and rash, with: myalgia, headache, arthralgia, or conjunctivitis	NR	11 (0–16)	RT-PCR	14^n^	100%	93%	29%	14%	29%	21%	NR	NR	0%
Cano and Esquivel (2018) [46]	Panama	2016–2017	Pediatric hospital-based surveillance system	Suspected ZIKV cases	29%	8 (3)^l^	RT-PCR	9^o^	29%^p^	86%	NR	NR	57%	43%	NR	29%	^q^
Salgado et al., (2019) [47]	Colombia	2016–2017	Hospital-based neurosurveillance system	Encephalitis cases	NR	NR (1 month-14y)^r^	RT-PCR ± IgM and/or IgG	6	50%	83%	33%	NR	NR	67%	NR	^s^	100%

CSTE: Council of state and territorial epidemiologists, GI: Gastro-intestinal, Ig: Immunoglobulin, NR: not reported, RT-PCR: Reverse transcription polymerase chain reaction, URT: Upper respiratory tract, WHO: World health organization, ZIKV: Zika virus

a The percentage reflects the highest reported frequency of the following URT symptoms: pharyngitis, pharyngeal erythema, rhinorrhea, cough, and congestion.

b The percentage reflects the highest reported frequency of the following GI symptoms: nausea, diarrhea, vomiting, abdominal pain.

c The case definition is based on fever and at least one of the following symptoms: nonpurulent conjunctivitis, headache, rash, pruritus or arthralgia, with no known alternate etiology. After December 2015, the definition was revised to include fever plus rash and at least one of the symptoms listed previously, myalgia, or malaise.

d The serological algorithm is based on acute and convalescent serum antibodies measured with DENV and ZIKV Inhibition enzyme-linked immunosorbent assays (iELISAs) and IgM-capture ELISAs as well as measurement of antibodies in post-infection healthy annual sample using the ZIKV NS1 blockade-of-binding (BOB) ELISA.

e CSTE definition: Any person with a clinically compatible illness for ZIKV infection that includes one or more symptoms of acute fever (reported or measured), rash, arthralgia, or conjunctivitis; OR Guillain-Barré syndrome or other neurologic manifestations; AND potential ZIKV exposure; OR Any person with laboratory evidence of recent ZIKV infection as indicated by: culture of ZIKV from blood, body fluid, or tissue demonstration of ZIKV-specific antigen or RNA in serum, cerebrospinal fluid (CSF), tissue, or other specimen (e.g., amniotic fluid, umbilical cord blood, urine, semen, saliva) ZIKV-specific immunoglobulin M (IgM) antibodies in CSF or serum[36]

f Symptoms were described in 140 out of 141 children with confirmed ZIKV infection.

g Percentage of children with any respiratory symptoms, here defined as: sore throat, cough.

h Percentage of children with any gastrointestinal symptoms, here defined as: nausea, vomiting, diarrhea, abdominal pain.

i Florida state testing criteria (for Miami-Dade County): presence of 2 of 4 main symptoms (rash, fever, joint pain, conjunctivitis) and recent travel history to an area outside of the United States experiencing ZIKV activity, or in the absence of travel outside of Miami-Dade County, presence of 3 of 4 main symptoms

j Sex and age are reported for the full case series (N = 33), which included 2 asymptomatic cases.

k WHO definition of possible ZIKV infection used in this study: erythematous eruption with or without fever and at least one of the following symptoms, arthralgia, arthritis or non-purulent conjunctivitis

l Mean age (SD)

m Symptom was described in 28 out of 29 children with confirmed ZIKV infection.

n Symptoms were described in 14 out of 25 children with confirmed ZIKV infection.

o Symptoms were described in 7 and GBS was reported in 2 out of a total of 12 children with confirmed ZIKV infection.

p 29% reflects the frequency of exanthem; 14% were reported to have petechiae.

q GBS was reported in two children with RT-PCR-confirmed ZIKV, but no frequency estimates were provided.

r The age range is reported for the full series of encephalitis cases (N = 20), which included 13 cases that were not confirmed to be ZIKV-related and 1 case of ZIKV/DENV co-infection.

s Intense vomiting was reported, but frequency estimates were unclear.

In all but one study, confirmatory testing for ZIKV was performed if cases presented with at least one common sign or symptom of ZIKV infection. In the remaining study, confirmatory testing was performed if cases presented with signs of encephalitis [47]. Confirmation of ZIKV infections was performed exclusively using laboratory assays in all except one study [40], which made use of a variety of patient records to ascertain ZIKV diagnosis based on CSTE criteria. These records included clinician reporting, laboratory reporting, death certificates, birth certificates and electronic medical records [36, 40]. Four studies, from Guadeloupe, the United States of America, and Colombia reported testing for concomitant infection by other arboviruses (dengue +/- chikungunya) [43, 45, 47, 49], although the testing method used was only reported in three studies [43, 45, 47].

Of the ten studies, nine [39–42, 44, 46, 47, 49] reported on the presence of common signs and symptoms of ZIKV infections [17], while the remaining study reported only on the presence of neurological disorders [43]. The most frequently reported ZIKV symptoms among confirmed pediatric ZIKV cases were rash (prevalence range: 29–100%), fever (55–99%), conjunctivital involvement (11–64%), arthralgia (14–48%), myalgia (6–57%), and headache (21–50%) (Table 1). Upper respiratory tract symptoms were reported in four studies (21–38% of cases), and gastro-intestinal symptoms were reported in four studies (2–29% of cases).

Neurological signs were not commonly detected in pediatric ZIKV patients. Only three out of seven population-based studies reporting on the presence or absence of neurological complications reported the presence of neurological signs [43, 46, 47]. One case series using retrospective surveillance system data from a children’s hospital in Panama, identified 2 RT-PCR-confirmed ZIKV infections among 12 GBS cases [46]. In a second hospital-based case series of pediatric encephalitis cases in Colombia, six cases had confirmed ZIKV mono-infections [47]. The median duration of encephalitis was five days, and additional reported symptoms included reduced responsiveness to analgesics, dehydration due to intense vomiting, and seizures in two patients. Neither of these two studies provided estimates of the frequency of the neurological complications among the general population of pediatric ZIKV cases [46, 47]. In the third and largest case series, a 2015–2016 Colombian study, 18,576 postnatal pediatric ZIKV cases were reported, of whom 1207 children had a laboratory-confirmed diagnosis of ZIKV infection [43]. Among the 18,576 possible cases of ZIKV, 96 cases with neurological conditions were reported, including 66 cases of polyneuropathy of which 40 cases had GBS, 17 cases of viral encephalitis, eight cases of demyelinating diseases, four cases of inflammatory diseases of the central nervous system, and one case classified as other neurological disorder.

Among the 1207 laboratory-confirmed cases, 1% (12/1207 cases) developed neurological complications, the details of which were not provided. Further, no information was available regarding any evaluation of neurodevelopment in these cases. Additionally, of the 40 ZIKV cases with a reported diagnosis of GBS, confirmatory laboratory testing appeared to be available for only two fatal cases (one case was positive for ZIKV, the other negative).

The same study also provided the only reports of mortality in pediatric ZIKV patients among case series. Study authors reported the death of six patients with confirmed ZIKV infection in their cohort (an estimated 0.5% of confirmed ZIKV cases) [43]. Further investigation into cause of death, which was available for three patients, revealed that, for two patients, death was attributed to acute myeloid leukemia and bacterial meningitis, respectively. For the final patient, death was attributed to complications of GBS, secondary to ZIKV infection.

Three studies investigated the prevalence of ZIKV signs and symptoms by age. The first study, led by Read and colleagues in Puerto Rico in 2016, presented the details of ZIKV-associated signs and symptoms in age groups <1y, 1-4y, 5-9y and 10-18y (S2 Fig) [44]. Objective clinical signs (fever, rash and erythema) were present among cases at a similarly high prevalence across age groups, whereas subjective symptoms such as headache, eye pain, arthralgia, myalgia and bone pain were three to nine times more prevalent in the oldest age group compared to the youngest age groups (10-18y *vs*. <1y or 1-4y). The prevalence of conjunctivitis as a symptom of ZIKV infection also appeared to increase with age, whereas the prevalence of cough appeared to decrease. Irritability was a symptom primarily prevalent in younger participants, with reported irritability among 56% of participants aged <1y, compared to 17 to 23% among other age groups. However, these age trends were only reported to be statistically significant for headache and irritability (p-value<0.05).

In the second study, a surveillance-based study in 10 states in the United States of America by Lindsey and colleagues, the details of ZIKV-associated signs and symptoms in children were presented in age groups 1-11y and 12-17y and compared to the frequency in adults (S3 Fig) [45]. Patent signs, such as rash and conjunctivitis, were present among cases at a similarly high prevalence between the two age groups, whereas fever, arthralgia and myalgia were more common in older children (12-17y) than younger children (1-11y) (p-value<0.05 for all). Arthralgia, arthritis, edema, and myalgia were less common in children overall compared to adults (p-value<0.05 for all). None of the children in this study were reported to experience neurological complications.

In the third study, a cohort study based in Nicaragua, Burger-Calderon and colleagues also noted that the clinical presentation of Zika virus infection differed across pediatric ages [39]. Among the 556 participants with confirmed ZIKV disease, older cases presented more frequently with arthralgia, myalgia, and headache. Of note, identification of cases based on the WHO and PAHO case definitions alone would have led to missed diagnoses of more than two-thirds of laboratory-confirmed symptomatic ZIKV infections in this cohort. However, the sensitivity of both the WHO and the PAHO case definitions increased with age (11% to 56% and 6% to 37%, respectively).

### Case reports

Twenty-two case reports from across 12 countries and territories described 40 pediatric cases of ZIKV (Fig 1, Table 2). Eleven cases (28%) were aged between 6 months and 5 years, 8 cases (20%) were aged 6 to 10 years, 19 cases (48%) were between 11 and 16 years, and 2 cases (5%) were less than 15 years but with an unspecified age. ZIKV infection was laboratory-confirmed by RT-PCR in 88% of cases (n = 35), and serology in 28% of cases (n = 11). Thirty-five cases (88%) underwent further testing for potential co-infections. Overall, the quality of the case reports varied from good (59% of studies) to poor (27% of studies) based on the Murad, et al., criteria (S3 Table) [38].

**Table 2:** Summary of case reports on postnatal ZIKV infection in children and adolescents, ordered by child age.

Author (year)	Location	Study year	Sex	Age (y)	ZIKV diagnostic test		Other pathogens tested (negative)	Common signs or symptoms						Additional signs or symptoms			Complications / Outcome	Co- morbidities
					RT-PCR	Serology		Rash	Fever	Conjunctivital Involvement	Arthralgia	Myalgia	Headache	URT symptoms	GI symptoms	Others		
Li et al., (2017) [23]	Singapore	2016	F	6m	+ ^2^	NR	CHIKV, DENV	+	+	-	-	-	-	-	NR	NR	Outcome: Full recovery	NR
Landais et al., (2017) [55]	Guadeloupe	NR	M	10m	+ ^1^	IgM -, IgG + (2^nd^ sample)	CMV, EV, HSV, VZV	+	+	+	NR	NR	NR	NR	NR	Right hemiparesis (Left MCA infarct)	Outcome: Partial motor recovery at 3 months. Walking acquired 13 months after disease onset	NR
Li et al., (2017) [23]	Singapore	2016	M	1y 3m	+ ^2^	NR	CHIKV, DENV	+	+	-	-	-	-	+	NR	NR	Outcome: Full recovery	NR
Brito Ferreira et al., (2017) [53]	Brazil	2014/ 2015	F	2	+ ^3^	IgM -	*Borrelia spp*., CMV, DENV, EBV, HIV, HSV, HTLV	+	+	NR	NR	NR	NR	NR	+	Itching, pruritus	Encephalitis Outcome: Motor sequelae in all four limbs and cognitive impairment at 3 months	NR
Sarmiento- Ospina et al., (2016) [50]	Colombia	2015	F	2	+ ^1^	NR	DENV	NR	NR	NR	NR	NR	NR	NR	NR	NR	Hepatomegaly, thrombocytopenia, bleeding diathesis Outcome: Anemic shock and death.	Probable acute leukemia
Li et al., (2017) [23]	Singapore	2016	F	2y 2m	+ ^1,^^2^	NR	CHIKV, DENV	+	+	-	-	-	-	-	NR	NR	Outcome: Full recovery	NR
Peralta-Aros et al. (2017) [78]	NR	2016	F	2y 6m	+	NR	NR	+	+	+	NR	NR	NR	NR	NR	Bone pain, weakness	Outcome: Full recovery with INS remission	Idiopathic nephrotic syndrome
Heang et al., (2012) [79]	Cambodia	2010	M	3	+	IgM +	CHIKV, DENV	-	+	NR	NR	NR	+	+	NR	NR	Outcome: Full recovery	NR
Li et al., (2017) [23]	Singapore	2016	F	3y 11m	+ ^1^^,^^2^	NR	CHIKV, DENV	+	+	-	-	-	-	+	NR	NR	Outcome: Full recovery	NR
Lednicky et al., (2016) [51]	Haiti	2014	M	4	+	NR	NR	NR	NR	NR	NR	NR	NR	NR	NR	Asymptomatic	NR	NR
Li et al., (2017) [23]	Singapore	2016	M	4y 1m	+ ^2^	NR	CHIKV, DENV	+	+	+	-	-	-	+	NR	NR	Outcome: Full recovery	NR
Wu et al. (2016) [80], Yin et al. (2016) [81]	China (TA-Venezuela)	2016	M	6	+ ^1^^,^^2^^,^^4^	NR	NR	+	+	+	NR	NR	NR	+	-	Lymphadenopathy	Outcome: Full recovery	NR
Lednicky et al., (2016) [51]	Haiti	2014	F	7	+	NR	NR	-	+	NR	NR	NR	NR	+	+	Anorexia	NR	NR
Peralta-Aros et al., (2017) [78]	NR	2016	M	7	+	NR	DENV	+	+	NR	NR	NR	NR	NR	NR	NR	Outcome: Full recovery with INS remission	Idiopathic nephrotic syndrome
Marinho et al., (2019) [56]	Brazil	2015	M	7	+^3^	PRNT+	CHIKV, DENV, EV, HSV-1, HSV-2, SLEV, TB, VZV, WNV	-	-	-	-	+	+	NR	+	Lower limb weakness and pain	Diffuse neurological manifestations, tonic-clonic seizures Outcome: Mild gait difficulty at discharge	NR
Duijster et al., (2016) [82]	Netherlands (TA-Dominica)	2016	M	8	+	NR	DENV	+	+	NR	NR	NR	+	NR	NR	Leucopenia	Outcome: Full recovery	NR
Wu et al. (2016) [80], Yin et al., (2016) [81]	China (TA-Venezuela)	2016	F	8	+ ^1^^,^^2^^,^^4^	NR	NR	+	+	+	NR	NR	NR	+	-	Severe leucopenia	Outcome: Full recovery	NR
Slavov et al., (2016) [83]	Brazil	2016	M	8	+ ^1^^,^^4^	NR	CHIKV, DENV	-	+	-	NR	+	+	NR	+	Retro-orbital pain, photo- and phonophobia, mesenteric adenitis.	Outcome: Full recovery	NR
Florescu et al., (2017) [84]	Romania (TA- French Guiana)	2016	M	10	+ ^2^	IgM +	DENV, *Rickettsia spp*., WNV, YF vaccine virus +	+	+	NR	NR	NR	NR	+	NR	Dysphagia, lymphadenopathy	NR	NR
Li et al., (2017) [23]	Singapore	2016	F	11	+ ^2^	NR	CHIKV, DENV	+	+	-	-	+	-	+	NR	NR	Outcome: Full recovery	NR
Li et al., (2017) [23]	Singapore	2016	F	11	+ ^2^	NR	CHIKV, DENV	+	+	+	-	-	-	-	NR	NR	Outcome: Full recovery	NR
Li et al., (2017) [23]	Singapore	2016	M	11	+ ^2^	NR	CHIKV, DENV	+	+	+	-	-	-	+	NR	NR	Outcome: Full recovery	NR
Li et al., (2017) [23]	Singapore	2016	F	11	+ ^2^	NR	CHIKV, DENV	+	+	-	-	-	+	-	NR	NR	Outcome: Full recovery	NR
Li et al., (2017) [23]	Singapore	2016	M	11	+ ^2^	NR	CHIKV, DENV	+	+	-	-	+	-	-	NR	NR	Outcome: Full recovery	NR
Olson et al., (1981) [85]	Indonesia	1977	M	12	NR	HI +	DENV, JEV, MVEV, TMUV	NR	+	NR	NR	NR	NR	NR	+	Malaise, dizziness	NR	NR
Olson et al., (1981) [85]	Indonesia	1978	F	12	NR	HI +	DENV, JEV, MVEV, TMUV	NR	+	NR	NR	NR	NR	NR	+	Malaise, anorexia, dizziness and hypotension.	NR	NR
Cleto et al., (2016) [54]	Brazil	NR	F	13	NR	IgM +	CHIKV, CMV, DENV, EBV, hepatitis A, B, and C, HSV, HTLV, rubella virus, *Toxoplasma gondii*	+	NR	NR	NR	NR	+	NR	NR	Migratory and intermittent paresthesia in hands, feet, and face	Peripheral Neuropathy Outcome: Regained ability to walk with support at discharge on day 15	NR
Li et al., (2017) [23]	Singapore	2016	M	14	+ ^2^	NR	CHIKV, DENV	+	-	-	-	-	+	+	NR	NR	Outcome: Full recovery	NR
Olson et al., (1981) [85]	Indonesia	1977	M	14	NR	HI +	DENV, JEV, MVEV, TMUV	NR	+	+	+	+	NR	NR	+	Dizziness, hematuria	NR	NR
Alejo-Cancho et al., (2016) [86]	Spain (TA- Honduras)	2016	F	<15	+ ^1^	IgM + IgG -	CHIKV, DENV	+	+	+	NR	NR	+	NR	NR	Chill	NR	NR
Alejo-Cancho et al., (2016) [86]	Spain (TA- Honduras)	2016	F	<15	+ ^1^	IgM—IgG -	CHIKV, DENV	+	+	+	NR	NR	+	NR	NR	Chill	NR	NR
Alera et al., (2012) [87]	Philippines	2012	M	15	+ ^1^	NR	CHIKV, DENV, JEV	-	+	+	NR	+	+	+	+	Anorexia, nausea	Outcome: Full recovery	NR
Arzuza-Ortega et al.,(2016) [58]	Colombia	2015	F	15	+ ^1^	NR	CHIKV, DENV, HIV-1/2, *Leptospira spp*., *Plasmodium spp*.	NR	+	NR	+	+	NR	NR	+	Retro-ocular pain, respiratory distress, jaundice	Hepatomegaly, splenomegaly Outcome: Death 5 days after disease onset	Sickle cell disease
Boyer Chammard et al., (2016) [88]	Guadeloupe	2016	F	15	+ ^1^^,^^2^	NR	DENV	+	NR	NR	NR	NR	NR	NR	NR	NR	Bleeding diathesis Outcome: full recovery	NR
Li et al., (2017) [23]	Singapore	2016	F	15	+ ^2^	NR	CHIKV, DENV	+	+	+	+	-	-	-	NR	NR	Outcome: Full recovery	NR
Paniz-Mondolfi et al., (2018) [57]	Venezuela	NR	F	15	+ ^1^	IgM+ IgG-	CHIKV, CMV, DENV, EBV, EV, HIV, HSV-1, HSV-2, VZV, PV, Rubella virus, *Treponema pallidum*, *Toxoplasma gondii*	+	+	NR	+	NR	NR	NR	NR	Malaise	Alice in Wonderland Syndrome (AWS) Outcome: Full recovery	NR
Mecharles et al., (2016) [52]	Guadeloupe	2016	F	15	+ ^1^^,^^2^^,^^3^	NR	*Borrelia spp*., CHIKV, CMV, DENV, EBV, HIV, HSV, HTLV, *Legionella spp*., *Mycoplasma pneumoniae*, *Treponema spp*., VZV	NR	-	+	NR	NR	+	NR	NR	Left arm and lower back pain. Paraesthesia	Acute myelitis. Outcome: Ability to walk recovered 1 month after hospital admission	NR
Li et al., (2017) [23]	Singapore	2016	M	16	+ ^2^	NR	CHIKV, DENV	+	+	-	-	-	-	+	NR	NR	Outcome: Full recovery	NR
Azevedo et al. (2016) [59]	Brazil	NR	F	16	+ ^1^	IgM -	CHIKV, DENV, hepatitis A, B, and C viruses, HIV-1/2, HTLV I/II, SLEV, *Treponema spp*., *Trypanosoma cruzi*, WNV, YFV	+	+	NR	NR	+	+	NR	NR	Malaise	Bleeding diathesis Outcome: death	Possible Evans syndrome
Olson et al., (1981) [85]	Indonesia	1977	F	16	NR	HI +	DENV, JEV, MVEV, TMUV	NR	+	NR	NR	NR	NR	NR	+	Malaise, chills, anorexia, dizziness and leg pain	NR	NR

- = test-negative or sign/symptom reported to be absent

+ = test-positive or sign/symptom reported to be present

CHIKV: Chikungunya virus, CMV: Cytomegalovirus, DENV: Dengue virus, EBV: Epstein-Barr Virus (human herpesvirus 4), EV: Enteroviruses, GBS: Guillain-Barré syndrome, HI: hemagglutination inhibition, HIV: Human Immunodeficiency virus, HSCT: hematopoietic stem cell transplant, HSV: Herpes simplex viruses, HTLV: human T-lymphotropic virus, Ig: immunoglobulin, JEV: Japanese encephalitis virus, MVEV: Murray valley encephalitis virus, NR: not reported, PV: Parvovirus, RT-PCR: Reverse transcription polymerase chain reaction, SLEV: Saint Louis encephalitis virus, TA: travel-associated, TB: *Mycobacterium tuberculosis*, TMUV: Tembusu virus, VZV: Varicella Zoster virus, WNV: West Nile virus, YF: Yellow Fever, ZIKV: Zika virus

1 Detected in serum

2 Detected in urine

3 Detected in cerebral spinal fluid

4 Detected in saliva

Individual symptoms of ZIKV infection were reported for 38 of the 40 patients. One study reported only on complications of ZIKV infection (i.e., hepatomegaly, thrombocytopenia, anemic shock) [50], and the remaining study reported only that the infection was asymptomatic [51]. The most common reported symptom was fever, which was present in 92% of reports (33/36 cases) mentioning presence or absence of fever in pediatric ZIKV cases (Table 2). Other common symptoms included rash (84%, 27/32 cases), conjunctivital involvement (54%, 13/24 cases), headache (52%, 12/23 cases), myalgia (42%, 8/19 cases), and arthralgia (24%, 4/17 cases). Upper respiratory tract symptoms were also present in 68% of cases (13/19) and GI symptoms in 83% of cases (10/12). Neurological complications were reported in 18% of cases (6/34) with information on the presence of neurologic signs. These included one case of acute myelitis [52], one case of encephalitis [53], one case of peripheral neuropathy [54], one case of left middle cerebral artery infarct with right hemiparesis [55], one case of seizures and diffuse neurological manifestations [56], and one case of Alice in Wonderland Syndrome [57].

Outcomes of ZIKV infection were available in 31 case reports. The majority of patients (74%, 23 cases) experienced a full recovery following ZIKV infection. As reported earlier, six cases suffered from neurological complications [52–57] and an additional three cases (ages 2, 15, and 16y) were reported to have died [50, 58, 59]. For all three fatal cases, there was evidence or likelihood of underlying co-morbidity (acute leukemia, sickle cell disease, Evans syndrome). Five of the six cases with neurological complications suffered from impaired motor function. Two cases (ages 13 and 15y) regained the ability to walk after two weeks and one month respectively [52, 54], one case was discharged from the hospital with mild gait difficulty [56], one case (age 10 mo) who had suffered from a stroke recovered partial motor function after 3 months [55], and the last case (age 2y) who suffered from encephalitis had major motor sequelae in all four limbs after three months [53]. No assessment of potential consequences on neurodevelopment was reported for any of the cases.

## Discussion

This review, which collated the evidence from 2543 pediatric ZIKV cases across 1 cohort study, 9 case series, and 22 case reports, summarizes the existing knowledge on the presentation and complications of symptomatic postnatal ZIKV infection in children and adolescents.

While the rates of symptomatic ZIKV infection in the general population have been studied extensively [16], there is limited information available regarding the proportion of symptomatic ZIKV infection in pediatric populations. Reliable estimates of the rate of asymptomatic ZIKV in pediatric populations are limited. The majority of investigations studying ZIKV infection in adolescent and pediatric populations use the presentation of ZIKV-like symptoms as a prerequisite for performing diagnostic testing, which precludes inclusion of asymptomatic ZIKV cases. Estimates of the prevalence of symptoms vary significantly between studies, from approximately 7% in Miami-Dade county (USA) [41] to 41% in Nicaragua [32] and 71% in French Polynesia [33]. Despite a lack of reliable estimates of the rates of symptomatic ZIKV infection in pediatric populations, findings from this review suggest that when symptomatic, pediatric ZIKV infection is primarily mild, with little evidence of severe complications or adverse outcomes. Unsurprisingly, the signs most commonly reported in pediatric cases, fever and rash, were also most frequently used to identify potential ZIKV cases. Findings from three studies describing symptoms by age suggested similar frequencies of signs, such as fever, rash and conjunctivitis across age groups, but lower frequencies of subjective pain-related symptoms, such as arthralgia and myalgia, in younger age groups, which is likely due to the difficulty of accurately identifying such self-reported symptoms in young children [39, 44, 45]. There was little evidence of severe outcomes among ZIKV patients, with only four studies (one case series [43] and three case reports [50, 58, 59]) reporting deaths among pediatric ZIKV patients. Further, for the majority of these events, there was evidence of underlying medical conditions (i.e., leukemia, sickle cell disease, and bacterial meningitis). With regards to the single population-based study reporting on ZIKV-associated mortality, the number of deaths reported in this study must be interpreted with caution as it is unclear whether confirmatory diagnosis of ZIKV infection was prioritized in deceased cases, potentially inflating the proportion of ZIKV-associated death. There was also limited evidence available regarding the prevalence and presentation of neurological complications of ZIKV infection, which is unsurprising given the rarity of these events and the small number of cases in population-based studies. Only the largest case series reported the presence of neurological complications, which were present in 1% of pediatric cases with a confirmed ZIKV diagnosis [43]. Cases of ZIKV-associated GBS were also reported in this study; however, details of GBS diagnosis and laboratory confirmation of ZIKV diagnosis were not available for all cases, limiting the reliability of the information.

Evaluating the rates and presentation of neurological complications in children and adolescents is of particular importance given the neurotropism of ZIKV and the vulnerability of young brains in development [34]. The strongest evidence for the neurotropic activity of ZIKV arises from prenatal exposure to the virus, which can lead to Congenital Zika Syndrome, one of the most severe manifestations being microcephaly [25]. Additional manifestations associated with congenital ZIKV infection that have been identified to date include epilepsy, motor abnormalities and bladder dysfunction [60, 61]. While the exact mechanism for the neurotropic activity of ZIKV remains unclear, a suggested mechanism is through the virus’s ability to halt the proliferation of neural progenitor cells and possibly induce cell death [62, 63]. Early postnatal ZIKV infection in rhesus macaques has also been shown to affect neurodevelopment, suggesting that ZIKV infection in infants may have potential long-term consequences [64]. However, no studies to date have assessed the potential consequences of non-congenital ZIKV infection on neurodevelopment in human children.

Based on the findings of the studies reported in this systematic review, the neurological consequences of postnatal exposure appear to be less severe compared to prenatal exposure to ZIKV. Studies showed little evidence of neurological complications in children and adolescents. However, an accurate assessment of the range and frequency of ZIKV-associated complications is limited by the paucity of data in this age group, and particularly in infants. Of note, among the six children with neurological complications, sequelae appeared to be more severe in the two youngest cases (≤2 years) [53, 55]. The two cases, of which one experienced an infarct and the other encephalitis, had not fully recovered from the neurological sequelae after three months [53, 55].

Beyond its effects on the central nervous system, ZIKV may also indirectly affect the peripheral nervous system through induced parainfective or post-infective autoimmune response [65, 66]. Increased numbers of GBS patients in ZIKV-endemic zones suggest that the virus may contribute to the pathogenesis of GBS. Using data on GBS and ZIKV cases from 11 locations between 2007 and 2017, a recent paper derived an estimate of 2 GBS cases per 10,000 ZIKV infections [67]. In conducting this review, we found little published evidence regarding the prevalence of GBS in pediatric ZIKV cases. Only two case series identified possible ZIKV-associated GBS cases in their study populations, but both were limited by the lack of information regarding confirmatory ZIKV diagnostic tests in these cases [43, 46].

There are strengths and limitations to this review. In order to include as many relevant studies as possible, we have used a broad search strategy and included French, Spanish and Portuguese terms given the geographical distribution of ZIKV. For the majority of cases, diagnosis of ZIKV infection was based on RT-PCR, the most reliable method of ZIKV diagnosis in regions with active flavivirus circulation. We have included both observational studies and case reports to provide a full summary of the existing evidence regarding postnatal ZIKV infections, but we note that the overall evidence base is limited as only one investigation was a cohort study with prospective ascertainment of infections. In addition, publication biases (i.e., systematic differences between published and unpublished evidence) remain a concern. First, observational studies without significant findings may be less represented in the appraised literature. Second, although useful for describing more severe and rare complications which would otherwise require large sample sizes to detect, case reports are typically biased towards unusual or severe disease presentations.

It is also important to note that the varying case definitions used to identify possible ZIKV cases in each study may limit the representativeness and exhaustiveness of the signs and symptoms reported in this review. In particular, studies focusing on identifying cases with rash [49], or fever [44] may have overreported these symptoms, leading to an overestimation of their prevalence in pediatric populations. Studies using a broader case definition and populations identified using cohort or national or county level surveillance data are likely to be more representative of symptomatic pediatric ZIKV infection. However, growing evidence suggests that standard case definitions, such as the PAHO and WHO definitions, have low sensitivity in pediatric populations and could lead especially to under-representation of the youngest children with ZIKV disease [39, 44, 45]. Furthermore, the signs and symptoms described in these studies may not be exhaustive, as atypical symptomatic ZIKV cases are unlikely to have been identified using routine surveillance systems.

The small sample sizes in the majority of case series, also limits our ability to assess the risk of ZIKV complications (in particular GBS) and mortality in children and adolescents given the low frequency of these outcomes, and incomplete ZIKV testing in studies reporting on these outcomes. Of particular note, despite Brazil being the epicenter of the 2015–2017 ZIKV outbreak [68], as laboratory confirmation of ZIKV infection was prioritized for pregnant women [69], no population-based pediatric studies with laboratory confirmation of ZIKV were available from Brazil.

While reliable diagnosis of ZIKV infection is often hampered by the limited availability of laboratory tests and the reliance on presentation of ZIKV-like symptoms, the majority of studies included in this review used RT-PCR. While RT-PCR is the most specific method for identifying acute ZIKV infection, the test has limited sensitivity and may misclassify ZIKV cases as false negatives, especially in patients with symptom onset more than 10 days before testing. Of note, two case reports relied exclusively on single serologic tests for confirmatory diagnosis (IgM and antibody hemagglutination) [54, 70], which have the potential to cross-react with other flaviviruses [71]. However, in both case reports, cases tested negative for dengue virus infection, which increases the confidence in the reliability of ZIKV diagnosis for these cases. Additionally, two case series reported on both laboratory-confirmed and probable ZIKV cases, hence the findings from these studies are less reliable. However, we note that in the study by Lindsey and colleagues (2020), only 11% of cases were probable, with the remaining 78% positive for ZIKV by RT-PCR and 11% by IgM with PRNT confirmation [45].

An important consideration when seeking to distinguish between non-congenital and congenital ZIKV infection is the age-range of the study population. This distinction is particularly difficult within the first month of life and is complicated by a lack of knowledge regarding viral persistence in congenitally infected neonates [22, 72]. However, there is only one study in this review which may have included children under one month old, as the age distribution of the 25 infants included in the study was not provided [44].

Due to increased urbanization and ecological shifts associated with climate change, the geographic distribution of key arthropod vectors (i.e., *Aedes Aegypti*, *Aedes Albopictus*) is expanding, putting new populations at risk of arbovirus infections [73, 74]. A better understanding of the short and long-term clinical consequences of ZIKV and other neurotropic arboviruses, including Dengue, Chikungunya, West Nile, and Japanese Encephalitis viruses [75–77], is essential to help establish appropriate control and prevention measures. Based on the evidence summarized in this review, we have proposed a list of outstanding questions as priorities for future research on ZIKV infections in children and adolescents:

- What is the full spectrum of clinical presentations associated with pediatric ZIKV infections? What factors (e.g., age, sex, prior flavivirus infection) influence the severity of ZIKV disease?
- What are the risks and spectrum of neurological complications associated with postnatal ZIKV infection in children and adolescents? Are neurological complications of ZIKV infection more severe in younger children? What is the risk of ZIKV-associated GBS in children and adolescents?
- What is the impact of non-congenital ZIKV infections, especially those in the first 1000 days of life, on pediatric neurodevelopment?

In conclusion, the available evidence identified in this review suggests that the presentation of ZIKV infection is primarily mild in children and adolescents, which may limit case ascertainment using standard clinical case definitions for ZIKV disease. Prospective follow-up of pediatric ZIKV cases using data linkage may help elucidate the long-term impacts of postnatal ZIKV infection in children and adolescents.

## Supporting information

S1 Table PRISMA checklist. (DOCX)

S2 Table Study quality assessment for case series using the criteria of Murad, et al., 2018 [38]. (DOCX)

S3 Table Study quality assessment for case reports using the criteria of Murad et al., 2018 [38]. (DOCX)

S1 Methods Literature search strategy: Search terms for each database used on 13 February 2020. (DOCX)

S1 Fig PRISMA flow diagram. (DOCX)

S2 Fig Prevalence of ZIKV-related signs and symptoms by age group reported by Read, et al., 2018 [44]^4^. (DOCX)

S3 Fig Prevalence of ZIKV-related signs and symptoms by age group reported by Lindsey, et al., 2020 [45]. (DOCX)
