# Supplementary material for: Postnatal symptomatic Zika virus infections in children and adolescents: A systematic review
Source: PLoS Negl Trop Dis. 2020 Oct 2;14(10):e0008612. doi: 10.1371/journal.pntd.0008612 (PMC7556487; doi:10.1371/journal.pntd.0008612)
Supplement: S1 Table — (DOCX) [file pntd.0008612.s001.docx]

S1 Table. PRISMA Checklist.

| **Section/topic** | **#** | **Checklist item** | **Reported on page #** |
| --- | --- | --- | --- |
| **TITLE** | | |  |
| Title | 1 | Identify the report as a systematic review, meta-analysis, or both. | p1 “Postnatal symptomatic Zika virus infections in children and adolescents: A systematic review” |
| **ABSTRACT** | | |  |
| Structured summary | 2 | Provide a structured summary including, as applicable: background; objectives; data sources; study eligibility criteria, participants, and interventions; study appraisal and synthesis methods; results; limitations; conclusions and implications of key findings; systematic review registration number. | p2 |
| **INTRODUCTION** | | |  |
| Rationale | 3 | Describe the rationale for the review in the context of what is already known. | p4 “While the presentation of ZIKV infection in adults is well-defined, there is limited information on the spectrum of clinical manifestations of ZIKV infections in children and adolescents (defined here as 0-18 years). This is an important gap to address as this age group comprises a substantial fraction of all ZIKV infections.” |
| Objectives | 4 | Provide an explicit statement of questions being addressed with reference to participants, interventions, comparisons, outcomes, and study design (PICOS). | P5 “This study aims to give an overview of the spectrum of clinical manifestations of non-congenital ZIKV infections in children and adolescents (0-18years) and highlight existing knowledge gaps for the scientific community to stimulate further research.” |
| **METHODS** | | |  |
| Protocol and registration | 5 | Indicate if a review protocol exists, if and where it can be accessed (e.g., Web address), and, if available, provide registration information including registration number. | P6 “We conducted this systematic review following a pre-defined research protocol registered in the PROSPERO database (CRD42019119260)” |
| Eligibility criteria | 6 | Specify study characteristics (e.g., PICOS, length of follow-up) and report characteristics (e.g., years considered, language, publication status) used as criteria for eligibility, giving rationale. | p6 “Eligible studies included cohort, cross-sectional, case series, and case report studies reporting on symptoms of postnatal ZIKV infection in children, aged 0 to 18 years of age, with a robust confirmation of ZIKV infection (i.e., laboratory confirmation by molecular or serologic test, or Council of State and Territorial Epidemiologists [CSTE] criteria^31^).” |
| Information sources | 7 | Describe all information sources (e.g., databases with dates of coverage, contact with study authors to identify additional studies) in the search and date last searched. | P6 “we performed a comprehensive literature search, with no date or language restrictions, using four databases (PubMed, Web of Science, LILACs, and EMBASE) from 1956 to 13 February 2020” |
| Search | 8 | Present full electronic search strategy for at least one database, including any limits used, such that it could be repeated. | Supplementary materials, eMethods 1 |
| Study selection | 9 | State the process for selecting studies (i.e., screening, eligibility, included in systematic review, and, if applicable, included in the meta-analysis). | p6 “Studies reporting on symptoms of congenital ZIKV infection or with evidence of a co-infection were excluded from the review.” |
| Data collection process | 10 | Describe method of data extraction from reports (e.g., piloted forms, independently, in duplicate) and any processes for obtaining and confirming data from investigators. | p6 “Three reviewers (AR, LL, and AV) performed the data extraction independently and cross-verified the results for accuracy and consistency.” |
| Data items | 11 | List and define all variables for which data were sought (e.g., PICOS, funding sources) and any assumptions and simplifications made. | P7 “Extracted data included information on: study author, study location, year, number of ZIKV cases, population source, ZIKV diagnostic testing, and differential diagnostic testing. Outcomes of interest extracted were the frequency of common signs and symptoms of ZIKV infection (i.e., rash, fever, conjunctivitis, and arthritis) and other signs and symptoms of ZIKV infection” |
| Risk of bias in individual studies | 12 | Describe methods used for assessing risk of bias of individual studies (including specification of whether this was done at the study or outcome level), and how this information is to be used in any data synthesis. | p7 “Two reviewers independently assessed study quality. The cohort study was assessed using the Oxford Centre for Evidence-based Medicine (OCEBM) Levels of Evidence, March 2009, which range from level 1 (highest) to 5 (lowest level of evidence). The case series and case reports were further evaluated using the study quality criteria proposed by Murad and colleagues (2018). Overall risk of study bias was based primarily on the assessment of participant exposure (i.e., ZIKV diagnosis) and outcomes (i.e., ZIKV signs, symptoms, and complications).” |
| Summary measures | 13 | State the principal summary measures (e.g., risk ratio, difference in means). | p7 “Outcomes of interest extracted were the frequency of (…) signs and symptoms of ZIKV infection” |
| Synthesis of results | 14 | Describe the methods of handling data and combining results of studies, if done, including measures of consistency (e.g., I^2^) for each meta-analysis. | Not relevant |
