# Supplementary material for: Postnatal symptomatic Zika virus infections in children and adolescents: A systematic review
Source: PLoS Negl Trop Dis. 2020 Oct 2;14(10):e0008612. doi: 10.1371/journal.pntd.0008612 (PMC7556487; doi:10.1371/journal.pntd.0008612)
Supplement: S2 Table — (DOCX) [file pntd.0008612.s002.docx]

S2 Table. Study quality assessment for case series using the criteria of Murad, et al., 2018.^38^

| Author (year) | 1. Does the patient(s) represent(s) the whole experience of the investigator (centre) or is the selection method unclear to the extent that other patients with similar presentation may not have been reported? | 2. Was the exposure adequately ascertained? | 3. Was the outcome adequately ascertained? | 4. Were other alternative causes that may explain the observation ruled out? | 5. Was there a challenge/rechallenge phenomenon? | 6. Was there a dose–response effect? | 7. Was follow-up long enough for outcomes to occur? | 8. Is the case(s) described with sufficient details to allow other investigators to replicate the research or to allow practitioners to make inferences related to their own practice? | **Overall rating** |
| --- | --- | --- | --- | --- | --- | --- | --- | --- | --- |
| Cano and Esquivel (2018) | Unclear | Unclear | Unclear | No | Not relevant | Not relevant | Unclear | No | **Poor** |
| Cordel et al., (2017) | Yes | Yes | Unclear | Yes | Not relevant | Not relevant | Unclear | Yes | **Fair** |
| Goodman et al., (2016) | Yes | Unclear | Unclear | No | Not relevant | Not relevant | Unclear | Unclear | **Poor** |
| Griffin et al., (2017) | Yes | Yes | Unclear | No | Not relevant | Not relevant | Unclear | Yes | **Fair** |
| Ho et al., (2017) | Yes | Yes | Unclear | No | Not relevant | Not relevant | Unclear | Unclear | **Fair** |
| Lindsey et al., (2020) | Yes | Yes | Yes | No | Not relevant | Not relevant | Unclear | Yes | **Fair** |
| Read et al., (2018) | Yes | Yes | Yes | No | Not relevant | Not relevant | Unclear | Yes | **Fair** |
| Salgado et al. (2019) | Yes | Yes | Yes | Yes | Not relevant | Not relevant | Yes | Yes | **Good** |
| Tolosa et al., (2017) | Yes | Yes | Unclear | Yes | Not relevant | Not relevant | Unclear | Unclear | **Poor** |
