# Supplementary material for: Postnatal symptomatic Zika virus infections in children and adolescents: A systematic review
Source: PLoS Negl Trop Dis. 2020 Oct 2;14(10):e0008612. doi: 10.1371/journal.pntd.0008612 (PMC7556487; doi:10.1371/journal.pntd.0008612)
Supplement: S3 Table — (DOCX) [file pntd.0008612.s003.docx]

S3 Table. Study quality assessment for case reports using the criteria of Murad et al., 2018.^38^

| Author (year) | 1. Does the patient(s) represent(s) the whole experience of the investigator (centre) or is the selection method unclear to the extent that other patients with similar presentation may not have been reported? | 2. Was the exposure adequately ascertained? | 3. Was the outcome adequately ascertained? | 4. Were other alternative causes that may explain the observation ruled out? | 5. Was there a challenge/rechallenge phenomenon? | 6. Was there a dose–response effect? | 7. Was follow-up long enough for outcomes to occur? | 8. Is the case(s) described with sufficient details to allow other investigators to replicate the research or to allow practitioners make inferences related to their own practice? | **Overall rating** |
| --- | --- | --- | --- | --- | --- | --- | --- | --- | --- |
| Alejo-Cancho et al., (2016) | Unclear | Yes | Yes | Yes | Not relevant | Not relevant | Unclear | No | **Poor** |
| Alera et al., (2012) | Yes | Yes | Yes | Yes | Not relevant | Not relevant | Yes | Yes | **Good** |
| Arzuza et al., (2016) | Unclear | Yes | Yes | Yes | Not relevant | Not relevant | Yes | Yes | **Good** |
| Azevedo et al. (2016) | Unclear | Yes | Yes | Yes | Not relevant | Not relevant | Yes | Yes | **Good** |
| Boyer Chammard et al., (2016) | Unclear | Yes | Yes | Yes | Not relevant | Not relevant | Yes | Unclear | **Fair** |
| Brito Ferreira et al., (2017) | Yes | Yes | Yes | Yes | Not relevant | Not relevant | Yes | Unclear | **Fair** |
| Cleto et al., (2016) | Unclear | Yes | Yes | Yes | Not relevant | Not relevant | Yes | Yes | **Good** |
| Duijster et al., (2016) | Yes | Yes | Yes | Yes | Not relevant | Not relevant | Yes | Unclear | **Good** |
| Florescu et al., (2017) | Yes | Yes | Yes | Yes | Not relevant | Not relevant | Yes | Yes | **Good** |
| Heang et al., (2012) | Yes | Yes | Unclear | Yes | Not relevant | Not relevant | Yes | No | **Poor** |
| Landais et al., (2017) | Unclear | Yes | Yes | Yes | Not relevant | Not relevant | Yes | Yes | **Good** |
| Lednicky et al., (2016) | Yes | Yes | Yes | Yes | Not relevant | Not relevant | Unclear | Yes | **Fair** |
| Li et al., (2017) | Yes | Yes | Yes | Yes | Not relevant | Not relevant | Yes | Yes | **Good** |
| Marinho et al., (2019) | Yes | Yes | Yes | Yes | Not relevant | Not relevant | Yes | Yes | **Good** |
| Mecharles et al., (2016) | Unclear | Yes | Yes | Yes | Not relevant | Not relevant | Yes | Yes | **Good** |
| Olsoni et al., (1981) | Yes | Unclear | Yes | Yes | Not relevant | Not relevant | Unclear | Yes | **Poor** |
| Paniz-Mondolfi et al., (2018) | Yes | Yes | Yes | Yes | Not relevant | Not relevant | Yes | Yes | **Good** |
| Peralta-Aros et al., (2017) | Unclear | Yes | Yes | Yes | Not relevant | Not relevant | Yes | Yes | **Good** |
| Sarmiento- Ospina et al., (2016) | Unclear | Yes | Unclear | Yes | Not relevant | Not relevant | Yes | Unclear | **Poor** |
| Slavov et al., (2016) | Unclear | Yes | Yes | Yes | Not relevant | Not relevant | Yes | Yes | **Good** |
| Wu et al., (2016) | Yes | Yes | Yes | Unclear | Not relevant | Not relevant | Yes | Unclear | **Poor** |
| Yin et al., (2016) | Unclear | Yes | Yes | Unclear | Not relevant | Not relevant | Unclear | Unclear | **Poor** |
