# Supplementary material for: Postnatal symptomatic Zika virus infections in children and adolescents: A systematic review
Source: PLoS Negl Trop Dis. 2020 Oct 2;14(10):e0008612. doi: 10.1371/journal.pntd.0008612 (PMC7556487; doi:10.1371/journal.pntd.0008612)
Supplement: S1 Methods — (DOCX) [file pntd.0008612.s004.docx]

S1 Methods. Literature Search Strategy: Search terms for each database used on 13 February 2020.

**PubMed:**

(zikv OR ZIKV OR "ZIKV Virus"[Mesh] OR "ZIKV Virus Infection"[Mesh]) AND (Infant[MeSH] OR infant* OR bébé OR bebé OR bebê OR baby OR babies OR neonat* OR nouvea* OR newborn* OR Toddler* OR bambin OR criança OR Child[MeSH] OR child* OR children OR enfant OR niño OR kid or kids OR boy OR boys OR muchachos OR garcon* OR garoto OR menino OR meninos OR girl OR girls OR fille* OR menina OR meninas OR niña OR chicas OR infanc* OR enfance OR Pediatrics[MeSH] OR P?ediatric* OR paediatric* OR peadiatric* OR pediátrico OR pédiatrique OR prepubescen* OR Adolescent[MeSH] OR adoles* OR teen* OR Puberty[MeSH] OR pubert* OR pubescen* OR puberdade OR pubertad OR puberté OR nursery school* OR kindergar* OR guardería OR préscolaire OR preescolar OR "pré escola" OR preschool* OR primary school* OR school OR elementary school* OR schoolchild* OR school age* OR Schools[MeSH] OR écol* OR escola OR escuela OR Secondary school* OR High school* OR Highschool*)

**Web of Science:**

(zikv OR ZIKV OR "ZIKV Virus"[Mesh] OR "ZIKV Virus Infection"[Mesh]) AND (Infant[MeSH] OR infant* OR bébé OR bebé OR bebê OR baby OR babies OR neonat* OR nouvea* OR newborn* OR Toddler* OR bambin OR criança OR Child[MeSH] OR child* OR children OR enfant OR niño OR kid or kids OR boy OR boys OR muchachos OR garcon* OR garoto OR menino OR meninos OR girl OR girls OR fille* OR menina OR meninas OR niña OR chicas OR infanc* OR enfance OR Pediatrics[MeSH] OR P?ediatric* OR paediatric* OR peadiatric* OR pediátrico OR pédiatrique OR prepubescen* OR Adolescent[MeSH] OR adoles* OR teen* OR Puberty[MeSH] OR pubert* OR pubescen* OR puberdade OR pubertad OR puberté OR nursery school* OR kindergar* OR guardería OR préscolaire OR preescolar OR "pré escola" OR preschool* OR primary school* OR school OR elementary school* OR schoolchild* OR school age* OR Schools[MeSH] OR écol* OR escola OR escuela OR Secondary school* OR High school* OR Highschool*)

**LILACs:**

((tw:(ZIKV)) OR (tw:(zikv))) AND ((tw:(infant* )) OR (tw:(bébé )) OR (tw:( bebé )) OR (tw:( bebê )) OR (tw:(baby)) OR (tw:(babies)) OR (tw:(neonat*)) OR (tw:(nouvea*)) OR (tw:(newborn*)) OR (tw:(toddler*)) OR (tw:(bambin )) OR (tw:(criança)) OR (tw:(child*)) OR (tw:(children)) OR (tw:(enfant )) OR (tw:( niño)) OR (tw:(kid )) OR (tw:(kids )) OR (tw:(boy )) OR (tw:(boys)) OR (tw:( muchachos)) OR (tw:( garcon*)) OR (tw:( garoto )) OR (tw:( menino)) OR (tw:(meninos )) OR (tw:(girl)) OR (tw:(girls )) OR (tw:(fille* )) OR (tw:(menina )) OR (tw:(meninas)) OR (tw:(niña)) OR (tw:( chicas)) OR (tw:( infanc* )) OR (tw:(enfance )) OR (tw:(P?ediatric* )) OR (tw:(paediatric* )) OR (tw:(peadiatric)) OR (tw:( pediátrico)) OR (tw:(pédiatrique)) OR (tw:( prepubescen* )) OR (tw:(adoles* )) OR (tw:(teen* )) OR (tw:(pubert* )) OR (tw:(pubescen*)) OR (tw:(puberdade)) OR (tw:(pubertad)) OR (tw:(puberté )) OR (tw:(nursery school*)) OR (tw:(kindergar*)) OR (tw:(guardería)) OR (tw:( préscolaire)) OR (tw:(preescolar )) OR (tw:("pré escola" )) OR (tw:(preschool* )) OR (tw:( primary school* )) OR (tw:(school )) OR (tw:(elementary school*)) OR (tw:(schoolchild* )) OR (tw:(school age* )) OR (tw:(écol*)) OR (tw:(escola )) OR (tw:(escuela)) OR (tw:(Secondary school*)) OR (tw:( High school* )) OR (tw:(Highschool*)))

**EMBASE:**
(zikv OR ZIKV or expl: ”ZIKV virus”) AND (infant* OR bebe OR baby OR babies OR neonat* OR nouvea* OR newborn* OR Toddler* OR bambin OR crianca OR child* OR children OR enfant OR nino OR kid OR kids OR boy OR boys OR muchachos OR garcon* OR garoto OR menino OR meninos OR girl OR girls OR fille* OR menina OR meninas OR nina OR chicas OR infanc* OR enfance OR P?ediatric* OR paediatric* OR peadiatric* OR pediatrico OR pediatrique OR prepubescen* OR adoles* OR teen* OR pubert* OR pubescen* OR puberdade OR pubertad OR puberte OR nursery school* OR kindergar* OR guarderia OR prescolaire OR preescolar OR "pre escola" OR preschool* OR primary school* OR school OR elementary school* OR schoolchild* OR school age* OR ecol* OR escola OR escuela OR Secondary school* OR High school* OR Highschool*)
