# Supplementary material for: Postnatal symptomatic Zika virus infections in children and adolescents: A systematic review
Source: PLoS Negl Trop Dis. 2020 Oct 2;14(10):e0008612. doi: 10.1371/journal.pntd.0008612 (PMC7556487; doi:10.1371/journal.pntd.0008612)
Supplement: S1 Fig — (DOCX) [file pntd.0008612.s005.docx]

*S1 Figure. PRISMA flow diagram.*

Full-text articles excluded

(n = 188)

- 32 reviews and commentary
- 12 conference abstracts
- 53 no clinical signs, symptoms, or complications reported
- 56 different target population
- 6 no laboratory ZIKV testing
- 23 congenital ZIKV infection
- 7 co-infections

Full-text articles assessed for eligibility
(n = 220)

## Eligibility

## Included

Studies included in qualitative synthesis
(n = 32)

Records identified through database searching PubMed, Web of Science, LILACs, EMBASE
(n = 9440)

Records excluded
(n = 6394)

Records screened
(n = 6614)

Records after duplicates removed
(n = 6614)

Additional records identified through other sources
(n = 1)

## Identification

## Screening
