# Supplementary material for: Postnatal symptomatic Zika virus infections in children and adolescents: A systematic review
Source: PLoS Negl Trop Dis. 2020 Oct 2;14(10):e0008612. doi: 10.1371/journal.pntd.0008612 (PMC7556487; doi:10.1371/journal.pntd.0008612)
Supplement: S2 Fig — (DOCX) [file pntd.0008612.s006.docx]

S2 Figure. Prevalence of ZIKV-related signs and symptoms by age group reported by Read, et al., 2018.^44^

Reported prevalence of signs and symptoms are relative to the number of diagnosed children with ZIKV infection confirmed by RT-PCR within each age group. Age trends were only statistically significant for irritability and headache (p<0.05).
