# Supplementary material for: Postnatal symptomatic Zika virus infections in children and adolescents: A systematic review
Source: PLoS Negl Trop Dis. 2020 Oct 2;14(10):e0008612. doi: 10.1371/journal.pntd.0008612 (PMC7556487; doi:10.1371/journal.pntd.0008612)
Supplement: S3 Fig — (DOCX) [file pntd.0008612.s007.docx]

S3 Figure. Prevalence of ZIKV-related signs and symptoms by age group reported by Lindsey, et al., 2020.^45^

Reported prevalence of signs and symptoms are relative to the number of diagnosed children with ZIKV infection confirmed by RT-PCR within each age group (1-11y; n=50 and 12-17y; n=90). Differences between pediatric age groups were significant for fever, arthralgia, and myalgia (p<0.05). Differences between children and adults were significant for arthralgia, myalgia, arthritis, and edema (p<0.05).
